# Supplementary material for: Competitive advantage, relationship, and benefit: Primary and secondary influencing factors of supply chain collaboration in China’s advanced manufacturing industry
Source: PLoS One. 2023 Jun 6;18(6):e0285247. doi: 10.1371/journal.pone.0285247 (PMC10243641; doi:10.1371/journal.pone.0285247)
Supplement: Attachment — Submitted filename: Response to Reviewers.docx [file pone.0285247.s004.docx]

**Response to Editor**

**Comment 1:** Please review your reference list to ensure that it is complete and correct. If you have cited papers that have been retracted, please include the rationale for doing so in the manuscript text, or remove these references and replace them with relevant current references. Any changes to the reference list should be mentioned in the rebuttal letter that accompanies your revised manuscript. If you need to cite a retracted article, indicate the article’s retracted status in the References list and also include a citation and full reference for the retraction notice.

**Response 1:** We are very grateful for these suggestions. They enhance the rigor and persuasiveness of the article. So we checked the reference list very carefully. We found that we did not cite the retracted article. But we found the problem of double citations. Therefore, we deleted the repeated references. Finally, we reordered the references according to the order of the citations in the text. To highlight the revisions, we have marked the revisions in the manuscript in red font (L1016-1253 in red).

**Response to Reviewers**

**Reviewer #1:**

Thank you very much for your positive and complimentary comments on the content of our article. It is good to know that all your concerns are properly addressed and you have no more suggestions. This gives us very great encouragement and support. We will continue to work hard. Thank you again for your recognition！

**Reviewer #2:**

Thank you for your insightful comments and careful guidance. Now we have carefully analyzed and revised exactly according to your comments. With the help of previous published studies, we revised the sentences and redeveloped the research gap in Introduction section. After deleting duplicate references, we have adjusted the reference list according to the added citations. In addition, we add the main points, restate the paper's main idea, and show how statements of the paper were proven in the last paragraph of Introduction section. After receiving your valuable comments, the level of our article gets great improvement.

**Comment 1:** authors need to grasp good ideas on developing the research gap in the Introduction. The manuscript concerning the presentation of ideas shows improvement, but there are still some sentence structure changes and develop more clear research gap with the support of previous published studies in the domain of your study aim. This is a beautifully analyzed and written paper with an important message. I would recommend publication after the issues below are addressed.Paper is in its well form, for further, I suggest the following. Impact of Social Supply Chain Practices on Social Sustainability Performance in Manufacturing Firms, International Journal of Innovation and Sustainable Development, Vol. 13, No. 2, 2019. DOI: 10.1504/IJISD.2019.098996.

**Response 1:** Thank you sincerely for your suggestion. In response to your suggestions, we have carefully read the research gap in the Introduction section of your proposed paper. We found that the paper you presented provided very much previous published studies in the development of the research gap. This is in line with your request to support and develop a clearer research gap with past research. In addition, we found that the research gap can be followed by a research question and an elaboration of research implications. Thank you very much for suggesting this article. By reading Impact of Social Supply Chain Practices on Social Sustainability Performance in Manufacturing Firms, it became clear how the research gap was formed and the logic between the research gap and the rest of the introduction. Therefore, we analyzed the current state of the research based on the three theoretical innovations of the paper before the research question. This process focused on adding past studies. We found an idea in the article you presented that could provide support for our analysis, so we also cited the article you presented. Finally, we developed a clearer research gap. We have also embellished the language in this section. To highlight the revisions, we have marked the revisions in the manuscript in red font (red labeled L77-105).

**Comment 2:** The last paragraph of the Introduction must be done a summary (resume) of the paper, i.e., a clear idea about what will be studied in the paper. I could not identify this. The last paragraph's purpose in the Introduction section is to summarize the main points, restate the paper's main idea, and show how the paper statements were proven. I think the paper will considerably improve and be a highly cited article.

**Response 2:** Thank you very much for your constructive guidance. We have revised the section in order according to your suggestions. First, we added the main content of the article at the beginning of the last paragraph of the introduction. That is, what will be studied in this article. Secondly, in this paragraph, our previous introduction to each part of the article clearly expresses how the ideas of the article are proven. Therefore, we only add the formulation of hypotheses to make the content clearer. Finally, we add the main ideas/results of the article at the end of this paragraph. To highlight the changes, we have marked them in red in the manuscript (L143-162 in red).

**Comment 3:** -Check the citations and references (one by one) if there is any missing information. Citations and references must be 100% accurate according to the journal guidelines.-Moreover, please check all the references in the text carefully.

**Response 3:** Thank you very much for your careful guidance. Based on your suggestions, we have carefully checked citations and references to avoid missing information and to ensure that citations and references correspond. In addition, the format of citations and references in this article is strictly in accordance with the journal guidelines. Items 71 and 72 of the references cite web links. Since we see that the format of web links is not defined in the journal guidelines, and we have seen web links placed in the reference list in other articles. So we put the web links directly. In order to highlight the revisions, we have marked them in red in the manuscript (L1016-1253 in red).
