# Supplementary material for: Competitive advantage, relationship, and benefit: Primary and secondary influencing factors of supply chain collaboration in China’s advanced manufacturing industry
Source: PLoS One. 2023 Jun 6;18(6):e0285247. doi: 10.1371/journal.pone.0285247 (PMC10243641; doi:10.1371/journal.pone.0285247)
Supplement: Attachment — Submitted filename: Response to Reviewers.docx [file pone.0285247.s005.docx]

**Response to Editor**

Thank you very much for your approval of the content of our article. Now we have carefully revised the two figures you mentioned and the reference list based on your comments. We hope our modifications will be approved by you.

**Comment 1:** The first letter of the word in Figure1 should be capitalized.

**Response 1:** We are very grateful for the suggestion. We capitalize the first letter of each word in Figure 1 and place the redrawn Figure 1 in the manuscript. We believe that the manuscript is more in line with the journal's specifications with this modification(red labeled L424).

**Comment 2:** Please enhance the clarity of Figure 3.

**Response 2:** Thank you very much for your careful guidance. We redrew Figure 3 according to the image format required by the journal and placed it in the manuscript. The clarity of Figure 3 is effectively improved. In addition, files of Figures 1, 2, and 3, which conform to the journal's format requirements, were uploaded in the manuscript submission system separately again(red labeled L812).

**Comment 3:** Please review your reference list to ensure that it is complete and correct. If you have cited papers that have been retracted, please include the rationale for doing so in the manuscript text, or remove these references and replace them with relevant current references.

**Response 3:** Thank you sincerely for your suggestion. Once again, we carefully checked the reference list. We found errors in the writing of some names, so we immediately corrected these errors. We are sorry for these errors. In addition, we have not cited papers that have been retracted. To highlight the revisions, we have marked the revisions in the manuscript in red font (red labeled L1052, L1055, L1085, L1108, L1148, L1184, L1187, L1191).
